# Telomere-to-telomere genome of *Phoebe chekiangensis* reveals that age-dependent CHG hypomethylation promotes floral transition via MADS-box gene activation

**DOI:** 10.1093/hr/uhag171

**Published:** 2026-04-24

**Authors:** Qinglin Sun, Baoan Wang, Yuting Zhang, Chunlong Li, Xiao Han, Qi Yang, Zaikang Tong, Junhong Zhang

**Affiliations:** National Key Laboratory for Development and Utilization of Forest Food Resources, Zhejiang A&F University, Hangzhou 311300, China; Zhejiang Key Laboratory of Forest Genetics and Breeding, Zhejiang A&F University, Hangzhou 311300, China; National Key Laboratory for Development and Utilization of Forest Food Resources, Zhejiang A&F University, Hangzhou 311300, China; Zhejiang Key Laboratory of Forest Genetics and Breeding, Zhejiang A&F University, Hangzhou 311300, China; National Key Laboratory for Development and Utilization of Forest Food Resources, Zhejiang A&F University, Hangzhou 311300, China; Zhejiang Key Laboratory of Forest Genetics and Breeding, Zhejiang A&F University, Hangzhou 311300, China; National Key Laboratory for Germplasm Innovation & Utilization of Horticultural Crops, Huazhong Agricultural University, Wuhan 430070, China; National Key Laboratory for Development and Utilization of Forest Food Resources, Zhejiang A&F University, Hangzhou 311300, China; Zhejiang Key Laboratory of Forest Genetics and Breeding, Zhejiang A&F University, Hangzhou 311300, China; National Key Laboratory for Development and Utilization of Forest Food Resources, Zhejiang A&F University, Hangzhou 311300, China; Zhejiang Key Laboratory of Forest Genetics and Breeding, Zhejiang A&F University, Hangzhou 311300, China; National Key Laboratory for Development and Utilization of Forest Food Resources, Zhejiang A&F University, Hangzhou 311300, China; Zhejiang Key Laboratory of Forest Genetics and Breeding, Zhejiang A&F University, Hangzhou 311300, China; National Key Laboratory for Development and Utilization of Forest Food Resources, Zhejiang A&F University, Hangzhou 311300, China; Zhejiang Key Laboratory of Forest Genetics and Breeding, Zhejiang A&F University, Hangzhou 311300, China

## Abstract

*Phoebe* species are renowned for their highly valuable ‘golden thread’ timber; however, their protracted juvenile phase presents a significant obstacle to mechanistic investigations of floral induction. *Phoebe chekiangensis*, a rare early-flowering representative within this genus, provides a unique model system for dissecting the vegetative-to-reproductive phase transition. Nevertheless, the absence of a high-quality reference genome has severely hindered molecular insights into its developmental regulation. Here, we present the first telomere-to-telomere (T2T) genome assembly for *P. chekiangensis*, comprising two completely gap-free haplotypes with contig N50 values exceeding 65 Mb, base-level quality scores >36, and Long Terminal Repeat Assembly Index scores surpassing the gold standard threshold of 20. Approximately 29 000 genes were annotated per haplotype, supported by a BUSCO completeness score of >97%. Age-resolved transcriptomic landscapes identified two MADS-box transcription factors, *PcMADS5* (*AP1*-like) and *PcMADS19.1* (*SOC1*-like), as core activators of the floral transition. Both genes triggered precocious flowering when ectopically expressed in *Arabidopsis thaliana*. Whole-genome bisulfite sequencing revealed a progressive, age-dependent decline in CHG (where H is A, C, or T) DNA methylation, which was particularly pronounced at the *PcMADS19.1* locus. Notably, *DML1/2*, which mediate active DNA demethylation, were coordinately upregulated during the onset of reproductive growth. Chemical demethylation using 5-azacytidine further diminished CHG methylation and selectively enhanced *PcMADS19.1* expression, confirming a causal relationship between CHG hypomethylation and transcriptional activation. This work delivers the first chromosome-scale T2T genome within the genus *Phoebe* and uncovers CHG demethylation as a previously unrecognized epigenetic switch governing reproductive competence in woody perennials.

## Introduction

The transition from vegetative growth to reproductive maturity represents a pivotal developmental switch in plants, culminating in the formation of floral organs. In perennial woody species, this phase change is notoriously protracted, necessitating an extended period of vegetative growth prior to the acquisition of reproductive competence. For example, *Pinus tabulaeformis* exhibits a juvenile period of 6–7 years [1], *Eucalyptus nitens* requires a decade or more [2], and *Quercus rubra* takes 20–25 years to reach reproductive maturity [3]. Consequently, this prolonged juvenile phase in forest trees severely impedes traditional breeding efforts, thereby restricting opportunities for rapid genetic improvement and germplasm conservation [4, 5]. Flowering mechanisms have been extensively characterized in annual plants. In the model species *Arabidopsis thaliana*, flowering time is orchestrated by a complex network of interconnected pathways, primarily comprising the vernalization, autonomous, gibberellin, photoperiod, and age-dependent pathways [6]. These pathways converge on key floral integrators, such as *SUPPRESSOR OF OVEREXPRESSION OF CONSTANS 1* (*SOC1*), *FLOWERING LOCUS T* (*FT*), and *APETALA1* (*AP1*)-like transcription factors (TFs), forming a robust spatiotemporal regulatory network that integrates endogenous developmental signals and environmental cues to precisely dictate the timing of floral induction [7–9]. However, compared to the comprehensive paradigms established in annual models, the molecular mechanisms governing reproductive timing and phase transitions in long-lived woody species remain poorly understood.

Beyond transcriptional regulation, DNA methylation serves as a crucial epigenetic layer that modulates developmental transitions. In plants, DNA methylation dynamics are meticulously controlled by three primary mechanisms: *de novo* methylation, maintenance methylation, and active demethylation. *De novo* methylation is predominantly mediated by the RNA-directed DNA methylation (RdDM) pathway, which involves core components such as *RNA POLYMERASE IV* (*POL IV*), *RNA-DEPENDENT RNA POLYMERASE 2* (*RDR2*), *DICER-LIKE 3* (*DCL3*), *RNA POLYMERASE V* (*POL V*), *ARGONAUTE* (*AGO*), and *DOMAINS REARRANGED METHYLTRANSFERASE 2* (*DRM2*). The maintenance of these methylation marks across cell divisions is executed by the *METHYLTRANSFERASE 1* (*MET1*) and *CHROMOMETHYLASE* (*CMT*) families, alongside various chromatin remodelers. Conversely, active demethylation is catalyzed by DNA glycosylases, including *REPRESSOR OF SILENCING 1* (*ROS1*) and *DEMETER* (*DME*). Together, these interconnected pathways are coordinately regulated by a complex network of chromatin-associated factors and histone modifiers [10]. Age-associated methylation signatures have facilitated the development of an epigenetic clock in *Pinus taeda*, wherein CG and CHG (where H is A, C, or T) DNA methylation levels at developmentally and metabolically relevant loci progressively decline with advancing age [11]. Furthermore, DNA methylation profoundly influences upstream transcriptional networks, including the expression of microRNAs. In *Phyllostachys edulis*, a targeted reduction in CHH methylation leads to the upregulation of *SPL3f*, which subsequently enhances *SOC1c* expression to trigger the floral transition [12]. Collectively, these findings highlight DNA methylation as a robust biomarker for aging and developmental progression; however, the precise mechanisms governing its age-dependent dynamics and its specific role in conferring reproductive competence in woody perennials remain largely enigmatic.

**Figure 1:**
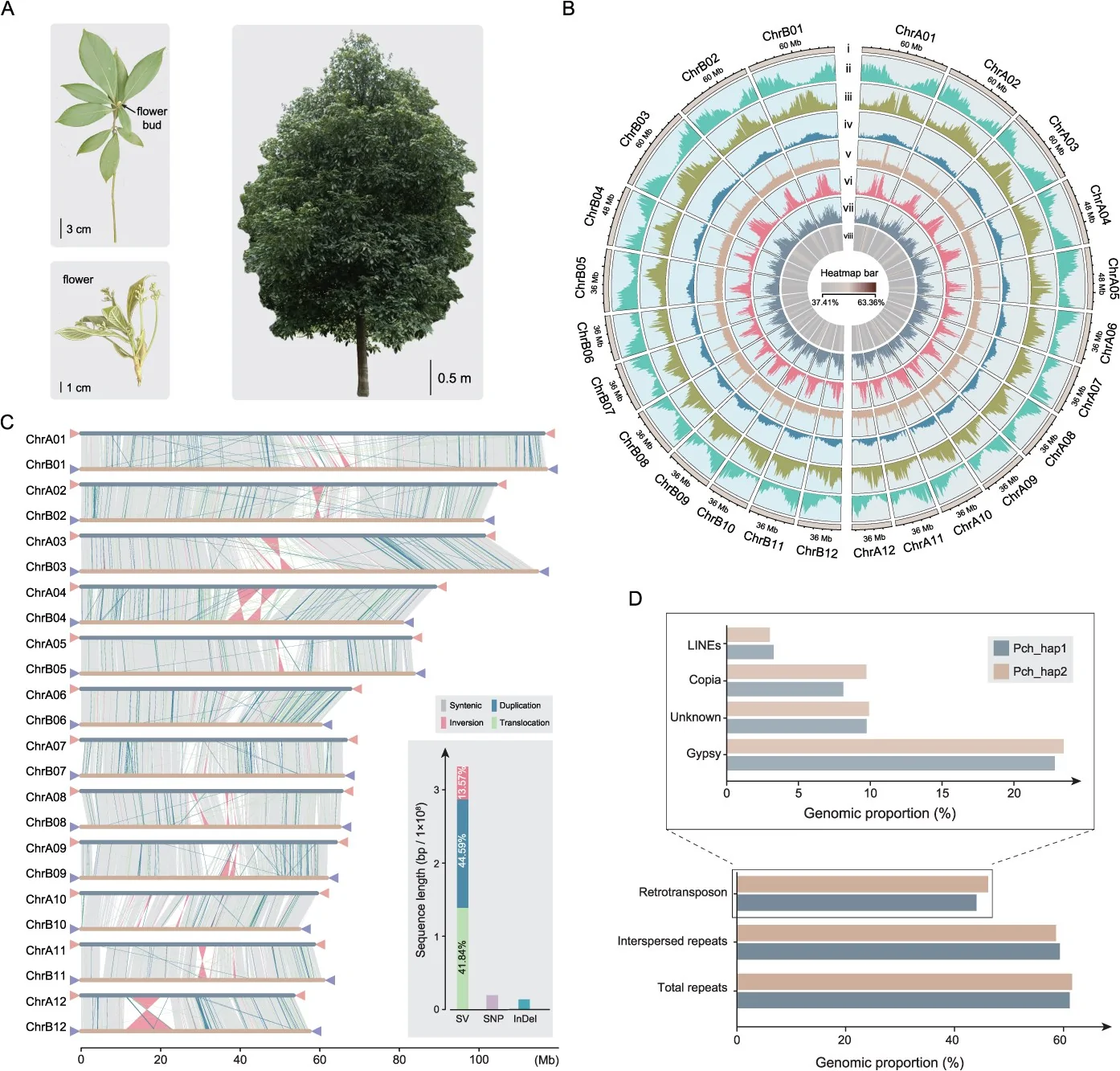
Morphology and genomic features of *P. chekiangensis.* (A) Morphology of the *P. chekiangensis* specimen used for genome assembly. The plant requires ~5 years to flower, with flowers and leaves emerging from the same bud. (B) Circos plot of the *P. chekiangensis* genome. Tracks from outer to inner: (i) chromosomes; (ii) HC gene density; (iii) LC gene density; (iv) repetitive sequence density; (v) LTR-unknown density; (vi) LTR-Gypsy density; (vii) LTR-Copia density; (viii) GC content (bin size = 1 Mb). (C) Collinearity between Pch_hap1 and Pch_hap2. Gray lines indicate syntenic blocks, highlighting extensive structural variations, primarily comprising duplications, inversions, and translocations. (D) Composition and abundance of repetitive sequences in Pch_hap1 and Pch_hap2, showing the predominance of LTR-RTs, including Gypsy, Copia, and Unknown types.

*Phoebe* species produce highly valued ‘golden thread’ wood, which is extensively utilized in premium furniture and holds substantial economic importance [13]. However, this genus comprises many species endemic to China that are highly endangered due to habitat loss and deforestation, with most surviving populations consisting of fewer than 70 individuals [14]. Members of this genus typically exhibit an extended vegetative phase that severely limits breeding and conservation efforts. In contrast, *Phoebe chekiangensis* possesses a relatively short juvenile period, initiating flowering after ~5 years. Notably, this species forms mixed buds at the branch terminals from which both flowers and leaves co-develop (Fig. 1A), making it an ideal model system for dissecting the vegetative-to-reproductive phase transition. Despite its biological significance, the lack of a high-quality reference genome has fundamentally hindered molecular research in this species. Here, we present the first telomere-to-telomere (T2T) genome assembly of *P. chekiangensis* alongside a single-base resolution DNA methylation map. Our analysis reveals that an age-dependent progressive decline in CHG methylation activates the *SOC1*-like gene *PcMADS19.1* and other floral promoters, thereby facilitating the transition to reproductive growth. Expression clustering further identified nine distinct transcriptional trajectories, with Cluster 7 genes specifically upregulated during reproductive onset. Notably, the active demethylase *DEMETER-LIKE/REPRESSOR OF SILENCING 1* (*DML1*) peaked at the 5-year transition point before declining, whereas *DML2* expression accumulated steadily with age. Functional validation utilizing the demethylating agent 5-azacytidine (5-azaC) alongside heterologous expression assays confirmed this epigenetic regulatory cascade. By integrating a complete genome assembly with age-stratified multi-omics data, we demonstrate that CHG demethylation functions as an age-dependent epigenetic switch that activates specific *MADS-box* TFs to trigger the floral transition. Ultimately, this study provides essential genomic resources for the conservation of an endangered species and elucidates a novel mechanism of age-dependent floral regulation in woody perennials, offering significant implications for accelerating future breeding programs.

## Results

### T2T genome assembly and unique telomeric features

Whole-genome sequencing was conducted on a 10-year-old *P. chekiangensis* individual. Initial Illumina short-read sequencing generated 46.5 Gb of data (~46.45× coverage), and a subsequent 17-mer-based genome survey estimated the genome size at 1001.97 Mb with a heterozygosity rate of 3.5% (Fig. S1). To construct a high-quality assembly, multiplatform sequencing data were systematically integrated, encompassing 90.8 Gb of PacBio HiFi reads (~90.71× coverage), 134.22 Gb of Oxford Nanopore ultra-long reads (~134.09× coverage), and 115.95 Gb of Hi-C data (~115.83× coverage) (Table S1). The diploid genome was *de novo* assembled utilizing the Verkko pipeline, yielding two distinct haplotypes: haplotype 1 (Pch_hap1; 923.00 Mb, contig N50 = 66.14 Mb, contig N90 = 54.24 Mb) and haplotype 2 (Pch_hap2; 917.64 Mb, contig N50 = 65.52 Mb, contig N90 = 54.88 Mb) (Table S2; Fig. 1B and S2). A comprehensive assessment of assembly quality via Benchmarking Universal Single-Copy Orthologs (BUSCO) analysis, utilizing the eudicot-conserved gene dataset, indicated exceptionally high completeness, with Pch_hap1 and Pch_hap2 scoring 97.33% and 97.12%, respectively (Fig. S3). Base-level accuracy was robust, exhibiting quality values (QVs) of 37.60 for Pch_hap1 and 36.79 for Pch_hap2, corresponding to a nucleotide-level accuracy exceeding 99.9%. Furthermore, the Long Terminal Repeat Assembly Index (LAI) reached 21.63 for Pch_hap1 and 22.64 for Pch_hap2 (Table 1), securely exceeding the gold standard threshold for reference genomes. Collectively, these metrics demonstrate the successful construction of the first high-accuracy, reliable T2T genome assembly for *P. chekiangensis*.

**Table 1:** Statistics and quality assessment of the Phoebe chekiangensis haplotype-resolved genome assembly.

Genomic feature	Pch_hap1	Pch_hap2
Assembly size (bp)	923,001,057	917,637,627
Contig N50 (bp)	66,135,712	65,521,137
Contig N50 number	6	6
Contig N90 (bp)	54,235,725	54,876,974
Contig N90 number	12	12
Number of gaps	0	0
Number of telomeres	24	24
Number of HC genes	28,739	28,818
Number of LC genes	70,771	72,487
GC content (%)	40.12	40
Repeat content (%)	61.14	61.57
Complete BUSCOs (%)	97.33	97.12
LAI	21.63	22.64
QV	37.60	36.79

Telomeric sequences were successfully identified across both haplotypes, resolving 24 complete chromosomes per haplotype (Fig. S4). The telomeric repeat units exhibited haplotype-specific variation (‘AAACCCT’ in Pch_hap1 and ‘AAATGAG’ in Pch_hap2), with both deviating from the canonical plant telomere motif (‘TTTAGGG’). Centromeric regions were characterized by high-density tandem repeats demonstrating substantial size variation, ranging from 476.83 kb to 4.30 Mb in Pch_hap1 and from 13.57 kb to 2.99 Mb in Pch_hap2 (Table S3; Fig. S5). Although the dominant centromeric monomer length across chromosomes was consistently 178 bp (Table S4), sequence alignments revealed notably low intragenomic conservation (36.12% identity). Subsequent phylogenetic analysis classified these monomers into two distinct clades, designated Class I and Class II, which contained both haplotype-specific and mixed clusters (Fig. S6). This structural organization suggests a complex evolutionary history involving simultaneous divergence and linkage between the two haplotypes.

### Genome characterization: gene structure, repetitive landscape, and evolution

Collinearity analysis between the two haplotypes revealed that approximately half of the genomic sequences exhibited substantial structural variation, encompassing 523.93 Mb in Pch_hap1 and 438.58 Mb in Pch_hap2. Large-scale structural variations, predominantly translocations and duplications, affected >300 Mb of the assembled sequence. These structural variations disrupted 6519 genes in Pch_hap1 and 6790 in Pch_hap2. Furthermore, the identification of an additional 150 Mb of noncollinear sequence between the haplotypes underscores a profound degree of structural divergence (Fig. 1C).

Genome-wide annotation of repetitive sequences identified 564.34 (61.14%) and 565.02 Mb (61.57%) of repetitive content in Pch_hap1 and Pch_hap2, respectively (Table S5). Interspersed repeats constituted the dominant class, particularly long terminal repeat retrotransposons (LTR-RTs) (including the *Gypsy*, *Copia*, and Unknown families), which accounted for 22.85%, 8.13%, and 9.74% of the Pch_hap1 genome, and 23.47%, 9.73%, and 9.91% of the Pch_hap2 genome (Fig. 1D). Sequence analysis demonstrated shared structural architectures across these repetitive classes. Notably, only 0.52%–1.93% of the elements maintained complete structural integrity by count, which sharply contrasts with the 10.22%–20.75% structural completeness observed when assessed by sequence length. This high fragmentation rate, coupled with molecular dating indicating that peak transposon activity occurred within the last 0.2 million years (Fig. S7), strongly supports a recent evolutionary burst of transposable element proliferation.

**Figure 2:**
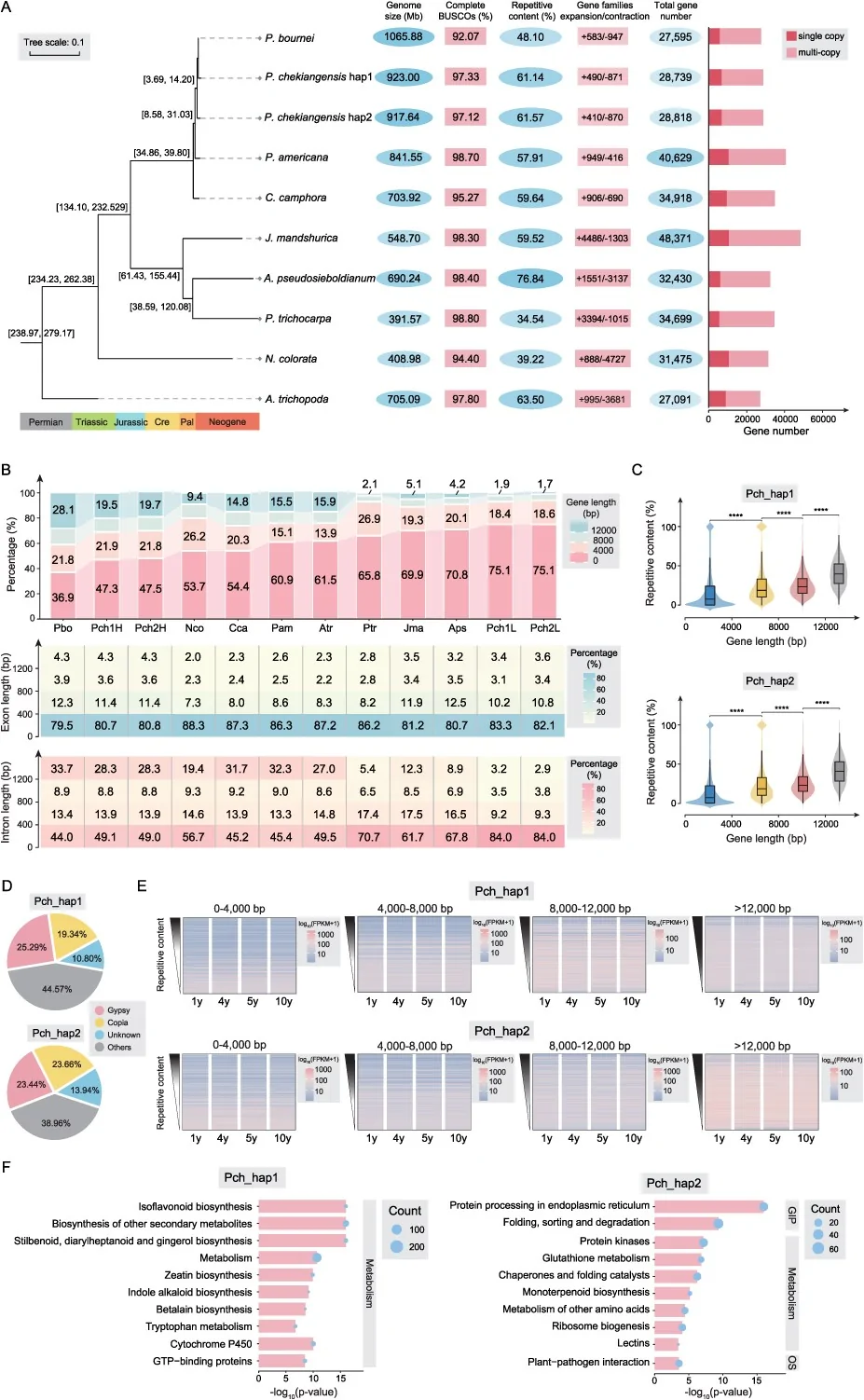
Evolutionary genomic analysis of *P. chekiangensis*. (A) Phylogenetic tree of nine angiosperm species based on 921 single-copy orthologs. Key genomic features are indicated, including genome size, BUSCOs, repetitive sequence content, gene family’s expansion/contraction, and gene number. (B) Gene length distribution across nine angiosperm species. Genes were grouped by length (0–4000, 4000–8000, 8000–12 000, and >12 000 bp). The proportion of genes longer than 12 000 bp clearly separates the gene sets into two groups. Intron length follows a similar trend, whereas exon length shows no significant difference across species. Species abbreviations: Pbo, *P. bournei*; Pch1H, *P. chekiangensis* hap1 HC genes; Pch2H, *P. chekiangensis* hap2 HC genes, Nco, *N. colorata*; Cca, *C. camphora*; Pam, *P. americana*; Atr, *A. trichopoda*; Ptr, *P. trichocarpa*; Jma, *J. mandshurica*; Aps, *A. pseudosieboldianum*; Pch1L, *P. chekiangensis* hap1 LC genes, Pch2L, *P. chekiangensis* hap2 LC genes. (C) Repetitive sequence content in genes of different lengths in *P. chekiangensis*. Repetitive content increases significantly with gene length. (D) Composition of repetitive sequences within *P. chekiangensis* genes. LTR-RTs constitute over half of the repetitive content, predominantly Gypsy, Copia, and Unknown types. (E) Correlation between repetitive sequence content and gene expression level in *P. chekiangensis*. Expression data are derived from 1-, 4-, 5-, and 10-year-old trees. Genes longer than 12 000 bp exhibit lower expression levels. Across all length groups, higher repetitive content correlates with reduced expression. (F) KEGG pathway enrichment analysis of expanded gene families in Pch_hap1 and Pch_hap2, revealing haplotype-specific functional biases. GIP, genetic information processing; OS, organismal systems.

To characterize the genomic evolution of this species, a phylogenetic tree was reconstructed utilizing 921 single-copy orthologs from nine high-quality plant genomes. This analysis confirmed that Lauraceae species, including *P. chekiangensis*, diverged later than basal angiosperms and form a sister clade to eudicots (Fig. 2A), fully consistent with established phylogenetic relationships. The annotated gene sets of *P. chekiangensis* were classified as high confidence (HC) if they exhibited active transcription (FPKM >1). This classification yielded 28 739 HC genes in Pch_hap1 and 28 818 in Pch_hap2 (Table S6). These gene counts are comparable to those of *Phoebe bournei* and *Amborella trichopoda*, although no significant correlation was observed among total gene number, average gene size, and repetitive sequence content across the compared species (Fig. 2A).

Comparative analysis further revealed distinct gene length distributions between the HC and low-confidence (LC) gene sets within the *P. chekiangensis* genome. Notably, the HC gene sets exhibited a higher proportion of exceptionally long genes (exceeding 12 000 bp), a structural feature shared with other Lauraceae species and basal angiosperms (Fig. 2B). While comprehensive gene structure analysis revealed no significant differences in exon length, exon number, or intron number among the evaluated gene sets, species characterized by long-gene enrichment consistently exhibited expanded intron lengths (Figs 2B and S8). Further investigation demonstrated that these extended gene lengths correlate robustly with high repetitive sequence content (Fig. 2C). Specifically, the previously detailed LTR-RTs constitute 55.43% and 61.04% of the total cumulative gene length in the Pch_hap1 HC and Pch_hap2 HC datasets, respectively (Fig. 2D). Moreover, global transcriptomic profiling indicated that gene expression levels are inversely proportional to internal repetitive sequence content (Fig. 2E). Collectively, these findings indicate that repetitive sequence insertions exert a substantial influence on both the structural architecture and the transcriptional activity of genes in *P. chekiangensis*. Gene family expansion and contraction patterns were highly concordant between the two haplotypes (Fig. 2A). However, KEGG pathway enrichment analysis revealed subtle haplotype-specific functional biases. Specifically, expanded gene families in Pch_hap1 are primarily involved in secondary metabolite biosynthesis, whereas those in Pch_hap2 additionally participate in protein metabolism and post-translational modifications (Fig. 2F).

### Transcriptome dynamics across development identify MADS-box genes associated with floral transition

**Figure 3:**
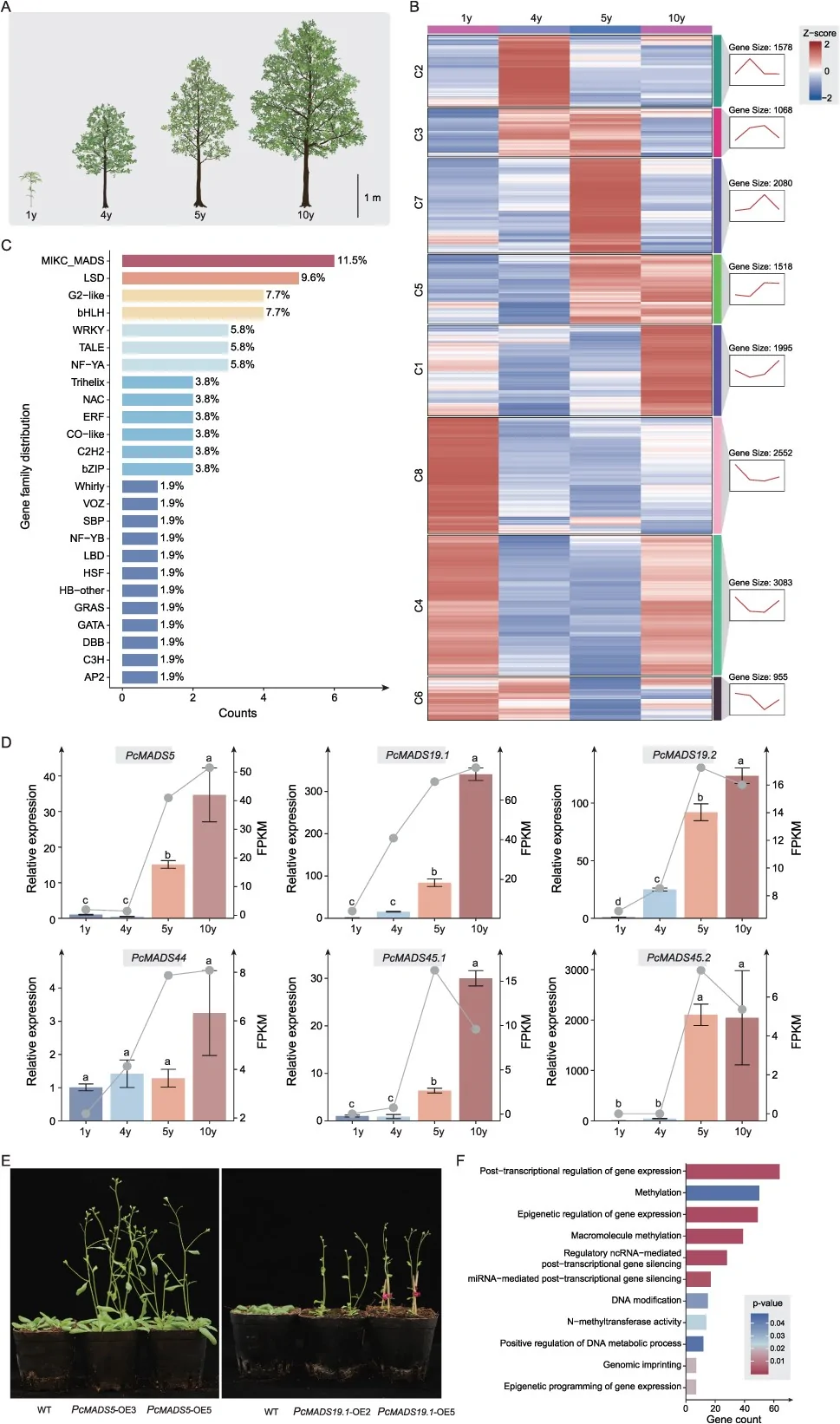
Age-dependent transcriptomic dynamics and functional roles of MADS-box genes in *P. chekiangensis* (A) Leaf samples collected from *P. chekiangensis* individuals at four developmental stages (1, 4, 5, and 10 years) for transcriptome sequencing. (B) Clustering analysis of differentially expressed genes across ages, highlighting eight optimal expression clusters. (C) Distribution of TF families within the upregulated expression module (Cluster 5), with notable enrichment of MADS-box genes. (D) Transcript levels (FPKM) and relative expression of upregulated MADS-box genes across samples, as determined by qRT-PCR. (E) Overexpression of *PcMADS5* and *PcMADS19.1* in *A. thaliana* promotes early flowering compared to WT controls. (F) GO enrichment terms for genes downregulated with age, showing significant enrichment of DNA methylation-related processes.

Utilizing the newly assembled high-quality genome as a reference, we conducted a comprehensive transcriptome analysis of leaf tissues across four distinct developmental stages (1-, 4-, 5-, and 10-year-old trees). All sequencing libraries met stringent quality thresholds, yielding >96% valid reads with Q30 scores exceeding 97% (Table S7). This robust dataset revealed pronounced age-dependent expression trajectories (Fig. 3A), facilitating the precise identification of genes orchestrating the vegetative-to-reproductive phase transition in *P. chekiangensis*. Subsequent differential expression profiling identified eight distinct transcriptional clusters based on the sum of squared errors (SSE) criterion (Fig. S9). Notably, genes within Cluster 5 exhibited rigorous developmental stage-specific regulation, characterized by basal expression levels during the juvenile phase (1 and 4 years) followed by significant upregulation upon reaching reproductive maturity (5 and 10 years) (Fig. 3B). Gene Ontology (GO) enrichment analysis indicated that Cluster 5 genes are predominantly associated with photosynthesis and chloroplast organization (Fig. S10; Table S8). Furthermore, KEGG pathway analysis demonstrated their critical involvement in energy metabolism (such as carotenoid biosynthesis and glutathione metabolism), alongside ribosome function and protein folding (Fig. S11; Table S9). Collectively, the robust activation of these functional categories likely maintains vital cellular homeostasis and meets the heightened metabolic demands during the energy-intensive vegetative-to-reproductive transition.

Detailed annotation revealed that Cluster 5 encompasses 32 TFs distributed across 25 families, featuring a significant enrichment of *MADS-box* genes (Fig. 3C). Quantitative real-time PCR (qRT-PCR) validation confirmed the age-dependent upregulation of five specific *MADS-box* genes during the reproductive stages. Among these, *PcMADS5* (belonging to the *AP1* subfamily) and *PcMADS19.1* (belonging to the *SOC1* subfamily) exhibited remarkably high transcript abundance (FPKM >20) (Fig. 3D), whereas the expression of control genes such as *PcMADS44* remained strictly basal. To rigorously assess their functional roles, we performed heterologous overexpression assays in *A. thaliana*. These experiments demonstrated that both *PcMADS5* and *PcMADS19.1* act as potent activators of flowering. Specifically, transgenic lines overexpressing *PcMADS19.1* exhibited highly precocious bolting (producing merely four rosette leaves prior to flowering), significantly accelerating the floral transition compared to *PcMADS5*-overexpressing lines (10 rosette leaves) and wild-type (WT) controls (14 rosette leaves) (Figs 3E and S12). In stark contrast, genes within Cluster 8 exhibited a consistent age-dependent downregulation trajectory. GO analysis revealed that this repressed module is significantly enriched for fundamental DNA modification processes, explicitly including DNA methylation (Fig. 3F; Table S10). This inverse regulatory pattern strongly implies a critical role for dynamic epigenetic reprogramming in governing the age-dependent developmental transition in this woody perennial.

### Identification and expression of DNA methylation pathway genes

**Figure 4:**
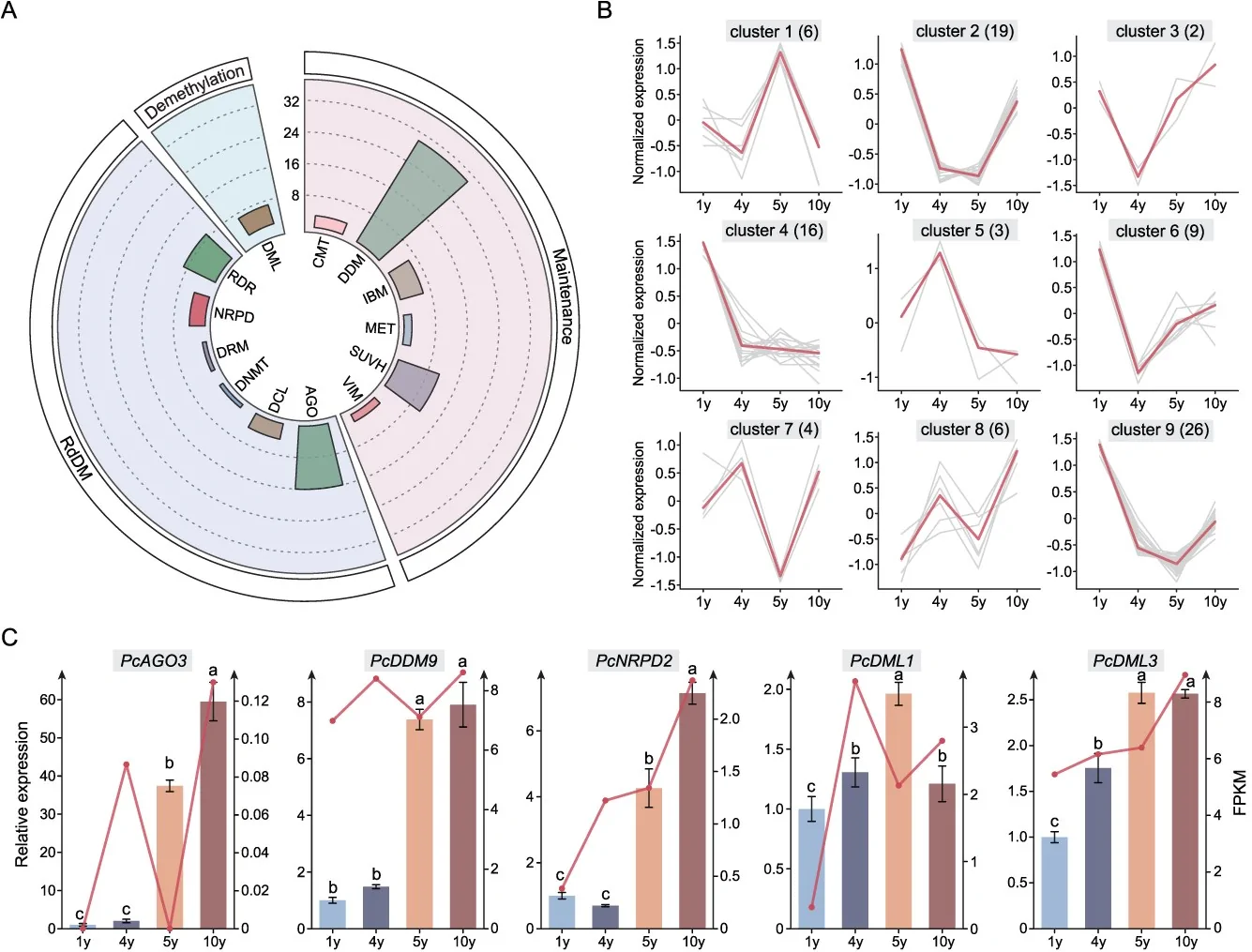
Identification and expression dynamics of DNA methylation regulatory components in *P. chekiangensis.* (A) Genomic distribution and classification of 94 identified epigenetic regulators involved in three core pathways: maintenance methylation, RNA-directed DNA methylation (RdDM), and active DNA demethylation. The radial bar chart illustrates the gene count within each functional subfamily, such as MET, CMT, AGO, and DML. (B) Unsupervised k-means clustering analysis of the 94 identified genes across four developmental stages (1, 4, 5, and 10 years), revealing nine distinct transcriptional trajectories. The number of genes assigned to each cluster is indicated in parentheses, and the central trend line represents the mean expression level for each cluster. (C) Quantitative validation of representative key regulators from the reproductive stage-associated module (Cluster 7). The bar charts represent relative expression levels determined by quantitative real-time PCR (qPCR) (mean ± SD), while the overlaid line plots represent transcript abundance measured in FPKM from RNA-seq data. Statistical significance is indicated by different lowercase letters (*P* < 0.05, one-way analysis of variance).

To elucidate the molecular basis of epigenetic regulation and active DNA demethylation in *P. chekiangensis*, we identified 94 core genetic components distributed across three major regulatory pathways (Figs 4A and S13, Table S11). The methylation maintenance apparatus represents the most extensive pathway, comprising 54 genes. This ensemble includes orthologs of the *MET* (2) and *CMT* (3) families, alongside newly identified components such as the *VARIANT IN METHYLATION* (*VIM*, 2), *SU(VAR)3 9 HOMOLOG* (*SUVH*, 11), *INCREASE IN BONSAI METHYLATION* (*IBM*, 6), and *DECREASED DNA METHYLATION* (*DDM*, 30) families.

The *RdDM* pathway consists of 35 members, featuring homologs of previously described *DCL* (4), *AGO* (16), *DRM* (1), and *RDR* (90 genes, as well as *DNA METHYLTRANSFERASE* (*DNMT*, 1) and *NUCLEAR RNA POLYMERASE D* (*NRPD*, 4). Additionally, five *DEMETER LIKE* (*DML*) orthologs were identified as the enzymatic basis for active DNA demethylation. Given the inherent complexity of woody plant genomes, this *DML* classification collectively encompasses members of both the highly conserved *ROS1* and *DME* subfamilies.

Unsupervised *k* means clustering of these 94 regulatory genes across the four developmental stages delineated nine distinct expression trajectories (Figs 4B and S14). Notably, genes assigned to *Cluster 7* exhibited a reproductive stage specific induction pattern, with transcript levels significantly elevated in 5- and 10-year-old trees compared to their juvenile counterparts. This critical cluster encompasses key regulatory nodes, including *PcAGO3*, *PcDDM9*, and *PcNRPD2*. Furthermore, quantitative transcript profiling confirmed that while *PcDML1* expression peaked sharply at the 5-year transition point before subsequently declining, *PcDML2* displayed a steady, age-dependent accumulation (Fig. 4C). Collectively, these robust expression trajectories are characterized by the coordinated upregulation of factors involved in both methylation maintenance and active DNA removal, highlighting a highly dynamic epigenetic reprogramming event during the transition to the reproductive stage.

### Genome-wide age-dependent dynamics of DNA methylation

**Figure 5:**
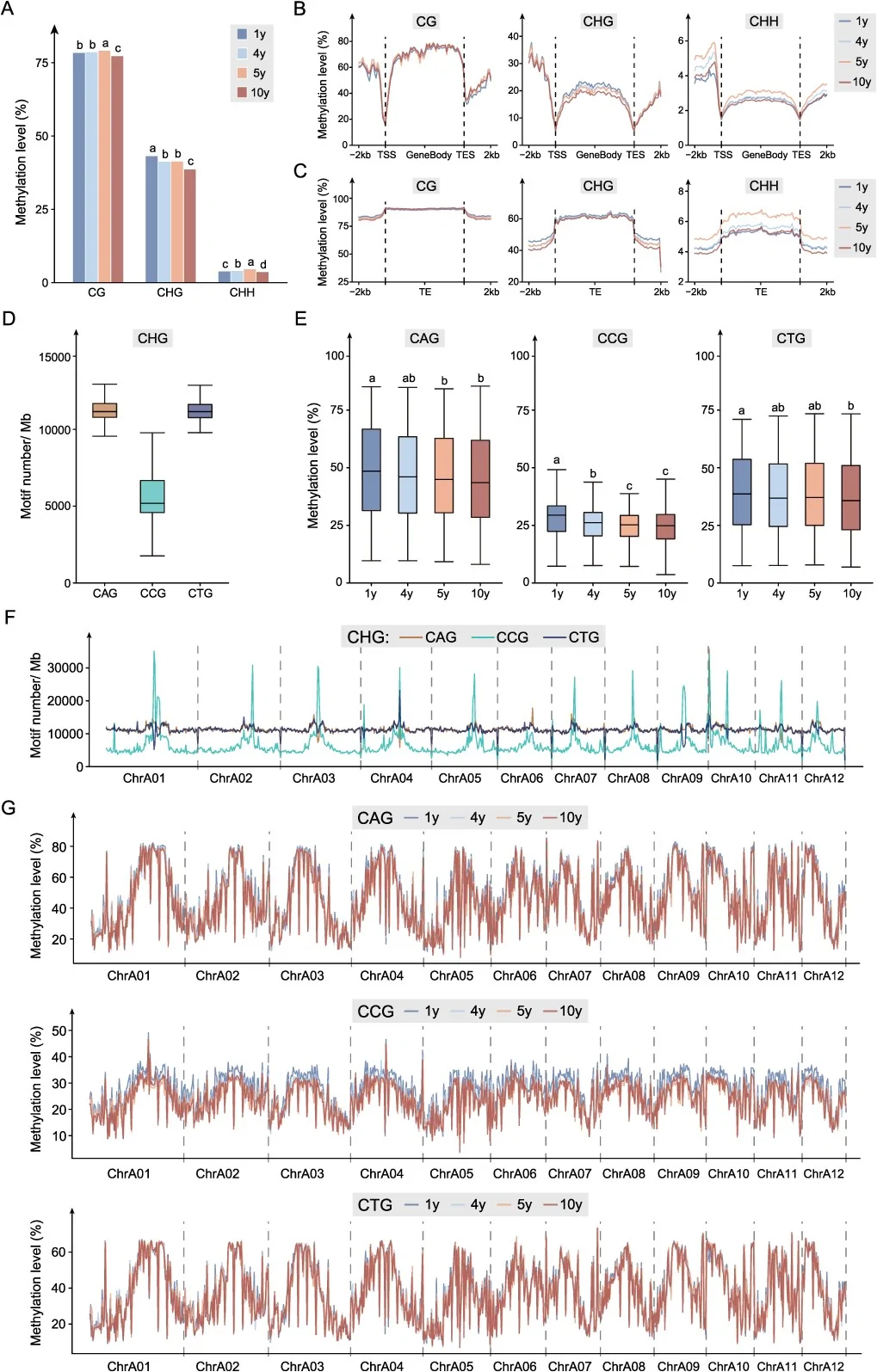
Genome-wide DNA methylation landscapes across developmental ages in *P. chekiangensis* (A) Global methylation levels of CG, CHG, and CHH contexts across four age groups (1, 4, 5, and 10 years). (B) Average DNA methylation levels across gene structures, showing methylation distribution from upstream to downstream regions for each age group. (C) Comparison of methylation levels between gene and TE regions for the three sequence contexts. (D) Methylation levels of CHG subcontexts (CAG, CCG, and CTG) across four age groups. (E) Chromosomal distribution of CHG subcontext site abundance in 1-Mb genomic bins. (F) Chromosomal distribution of CHG subcontext methylation levels in 1-Mb genomic bins across ages.

To elucidate the epigenetic regulation governing developmental phase transitions, whole-genome bisulfite sequencing (WGBS) was conducted on leaf tissues from four distinct age groups of *P. chekiangensis* (1-, 4-, 5-, and 10-year-old trees). High-coverage sequencing, exceeding 30× depth with >98% bisulfite conversion efficiency and 80% cytosine coverage, ensured highly reliable methylation profiling (Table S12; Fig. S15). Global genomic analysis revealed a progressive, age-dependent attenuation of CHG methylation, which decreased substantially from 37.42% in 1-year-old saplings to lower basal levels in subsequent reproductive stages. In stark contrast, global CG methylation remained consistently stable (77.42%), and CHH methylation exhibited only transient, nondirectional fluctuations (Fig. 5A; Table S13). Notably, this progressive CHG hypomethylation was predominantly localized within gene bodies (Fig. 5B), diverging fundamentally from the persistently stable methylation levels observed across transposable elements (TEs) (Fig. 5C).

To dissect these age-related methylation dynamics with higher resolution, CHG subcontexts (CAG, CCG, and CTG) were systematically analyzed across 1-Mb genomic bins. Quantitative assessment indicated that CAG and CTG sites averaged 11 000 loci per bin with highly stable distributions, whereas CCG sites averaged only 6566 loci per bin and displayed considerably greater spatial variability. Three distinct subcontext-specific methylation trajectories were identified through this comprehensive analysis (Fig. 5D; Table S14). Specifically, CCG methylation underwent a sharp reduction prior to the 4-year stage and subsequently stabilized. Furthermore, CAG methylation remained stable until Year 4 before initiating a distinct downward trend. Additionally, CTG methylation exhibited a steady and gradual reduction across the entire aging process (Fig. 5E; Table S15). In contrast, CG contexts maintained uniformly high methylation levels across all developmental stages, while CHH contexts exhibited stochastic variation that was completely uncoupled from chronological age (Figs S16 and S18).

Detailed chromosomal mapping demonstrated that CAG and CTG sites were generally more abundant than CCG sites genome-wide; however, a striking exception occurred within centromeric regions, where CCG density significantly exceeded the other subcontexts (Fig. 5F). Despite this pronounced physical enrichment of CCG motifs in centromeres, their localized methylation levels remained largely stable, with the exception of marginal increases observed on chromosomes 1 and 4. At the whole-chromosome scale, CAG and CTG methylation landscapes lacked clear age-dependent spatial patterns, whereas global CCG methylation exhibited a persistent, age-dependent decline (Fig. 5G). Paralleling the structural distribution of CHG subcontexts, CG sites were also physically enriched within centromeric boundaries but did not display correspondingly elevated methylation levels (Fig. S14). Conversely, CHH subcontexts were structurally less frequent in these regions yet exhibited paradoxically higher local methylation levels (Figs S17 and S19).

### Age-dependent hypomethylation activates the floral regulator *PcMADS19.1*

**Figure 6:**
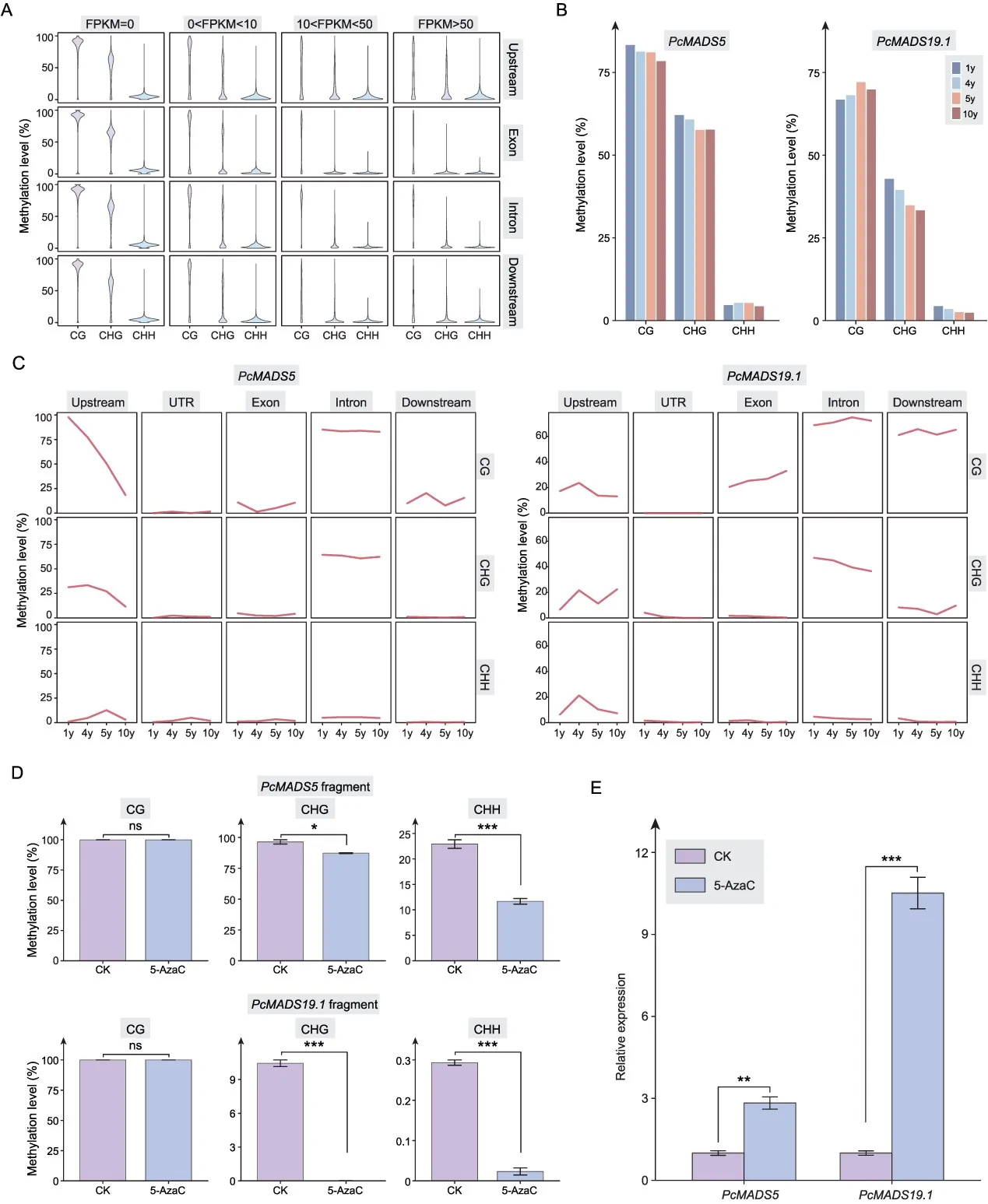
DNA methylation dynamics of MADS genes and their functional regulation in *P. chekiangensis*. (A) CG, CHG, and CHH methylation levels in different expression level genes. (B) Age-dependent changes in CG, CHG, and CHH methylation levels at the *PcMADS19* and *PcMADS5* locus across four developmental stages (1, 4, 5, and 10 years). (C) Feature-specific methylation distribution of *PcMADS19* and *PcMADS5*. (D) Bisulfite sequencing (BSP) results showing methylation changes in *PcMADS19* and *PcMADS5* after 5-azaC treatment in 10-year-old leaves. (E) Relative expression levels of *PcMADS19* and *PcMADS5* in control and 5-azaC-treated samples, as determined by qRT-PCR.

Globally, highly expressed genes exhibited correspondingly lower CHG methylation levels across the genome (Fig. 6A). At the locus-specific level, the *PcMADS5* locus experienced a slight decrease in CG methylation (from 83.51% to 78.58%) and CHG methylation (from 62.29% to 57.91%) during development, whereas its CHH methylation peaked at 5.48% in 5-year-old trees before subsequently declining. In contrast, the *PcMADS19.1* locus exhibited a marked, continuous decline in both CHG methylation (from 42.99% to 33.47%) and CHH methylation (from 4.53% to 2.52%), concomitant with a marginal increase in CG methylation (from 66.94% to 70.03%) (Fig. 6B; Table S16).

High-resolution, region-specific analysis further delineated these distinct epigenetic trajectories. The promoter region of *PcMADS5* demonstrated a dramatic reduction in CG methylation (from 97.56% to 18.40%) alongside a substantial decrease in CHG methylation (from 31.25% to 11.53%). Conversely, *PcMADS19.1* was characterized by significant CHG demethylation strictly within its transcribed regions, displaying marked reductions in both exonic (from 1.59% to 0.26%) and intronic (from 46.99% to 36.57%) intervals over time (Fig. 6C; Table S17).

To directly evaluate the functional impact of DNA methylation on the transcriptional activation of these candidate genes, vegetative branches of *P. chekiangensis* were exogenously treated with the DNA methyltransferase inhibitor 5-azaC for 15 days. Subsequent targeted bisulfite sequencing confirmed a significant reduction in methylation at both loci following treatment. Specifically, *PcMADS5* exhibited a moderate decrease in CHG methylation (from 96.33% to 86.29%) and CHH methylation (from 25.33% to 11.66%). Notably, *PcMADS19.1* demonstrated a much more pronounced depletion, with CHG methylation plummeting from 6.67% to 1.11% and CHH methylation being entirely eliminated (from 0.30% to 0%) (Fig. 6D; Table S18). Crucially, corresponding qRT-PCR profiling revealed that this chemical demethylation selectively and significantly upregulated *PcMADS19.1* expression, whereas *PcMADS5* transcript abundance remained largely unaffected (Fig. 6E). These results unequivocally confirm that the expression of the floral integrator *PcMADS19.1* is exquisitely sensitive to, and directly governed by, targeted DNA methylation dynamics.

## Discussion

*Phoebe chekiangensis* is a rare tree species endemic to southern China, highly valued for its exceptional ornamental qualities and premium timber [15]. Its protracted juvenile phase and limited capacity for natural regeneration present formidable challenges to both conservation and breeding efforts. Furthermore, the historical absence of a high-quality reference genome has severely constrained mechanistic studies elucidating its developmental regulation, adaptive evolution, and reproductive biology. In this study, we present the first gap-free, T2T genome assembly for this species. This comprehensive genomic resource provides an unprecedented foundation for investigating its complex genomic architecture, evolutionary history, and the molecular underpinnings of the vegetative-to-reproductive phase transition.

Repetitive sequences constitute fundamental components of plant genomes, substantially contributing to global genome structure, chromosome stability, gene duplication, and the evolutionary diversification of regulatory elements [16–18]. These elements play essential roles in maintaining telomere and centromere integrity; moreover, their insertion into genic regions can profoundly influence spatial gene regulation and drive phenotypic diversity [19–22]. In *P. chekiangensis*, the vast abundance of repetitive sequences, particularly those highly enriched within telomeric and centromeric boundaries, structurally supports chromosome stability while simultaneously providing a dynamic molecular basis for the extensive structural variation observed between haplotypes. Collectively, these genomic features underscore the central role of repetitive sequences in driving genome organization and adaptive evolution. Our high-resolution assembly establishes a robust foundation for future investigations into telomere and centromere biology, as well as the epigenetic regulation of key developmental genes.

A substantially expanded gene architecture represents a defining feature of the *P. chekiangensis* genome, a phenomenon primarily driven by the extensive insertion of LTR-RTs within intronic regions. Although exceptionally long genes can impose physical constraints on transcriptional efficiency and splicing precision [23–25], they concurrently promote genomic plasticity by supplying additional sequence space for structural variation and regulatory innovation [26, 27]. Comparative genomic analyses demonstrate that this intron expansion is highly conserved across the magnoliid clade, indicating an ancient evolutionary architectural trait that likely reflects deeply conserved regulatory mechanisms. In stark contrast, eudicot genomes typically exhibit considerably shorter gene lengths characterized by significantly fewer repetitive insertions. This fundamental dichotomy highlights pronounced lineage-specific trajectories in the evolution of gene architecture, further underscoring the substantial role of transposable elements in shaping both global genome structure and fine-scale transcriptional regulation.

The vegetative-to-reproductive phase transition represents a critical developmental milestone in the life cycle of trees, carrying profound implications for individual reproductive success, population-level ecological adaptation, and the macroscopic maintenance of forest structure [28–31]. This complex transition necessitates extensive tissue differentiation and the *de novo* development of reproductive organs. Furthermore, it requires the highly coordinated regulation of gene expression, metabolic networks, and chromatin states at the cellular level to ensure proper floral bud formation, subsequent flowering, and eventual fruiting [32–34]. At the molecular level, orchestrating this developmental shift relies heavily upon an intricate network of transcriptional regulators and dynamic epigenetic mechanisms, most notably DNA methylation [34–36]. Utilizing our high-quality *P. chekiangensis* genome as a reference, comprehensive transcriptomic analyses across successive developmental stages revealed that age-upregulated genes are predominantly enriched in photosynthesis, chloroplast organization, and fundamental energy metabolism. This metabolic reprogramming likely provides the necessary energetic foundation to support the high demands of flowering and fruiting. Crucially, several *MADS-box* TFs, including the *SOC1* homolog *PcMADS19.1* and the *AP1* subfamily member *PcMADS5*, were significantly upregulated, strongly implicating their core role in age-dependent floral regulation. Concurrently, essential DNA methylation regulatory genes (e.g. *DML*, *AGO*, *NRPD*, and *DDM*) exhibited a strict reproductive stage-specific upregulation. Specifically, *PcDML1* expression peaked sharply at the 5-year transition mark before declining, whereas *PcDML2* accumulated steadily with advancing age. These distinct trajectories reflect a highly dynamic epigenetic reprogramming landscape across progressive developmental stages. Coupled with the genomic expansion of the *CMT*, *SUVH*, and *DDM* gene families and the strict evolutionary conservation of RdDM components, these expression patterns indicate that precise epigenetic regulation plays an indispensable role in simultaneously safeguarding genome stability and orchestrating developmental precision.

Long-lived woody perennials have evolved to exploit dynamic CHG methylation as a sophisticated ‘developmental lock’ to enforce their characteristic extended juvenile phases and optimize the timing of reproductive onset [37–41]. The robust maintenance of this repressive epigenetic state is governed by a self-reinforcing feedback loop involving *CMT3* and histone H3 lysine 9 (H3K9) methyltransferases. Collectively, these components forge a restrictive chromatin environment that precludes the premature activation of floral promoters during the protracted vegetative phase [37, 42]. In *P. chekiangensis*, the transition to reproductive competence necessitates a strategic and progressive relaxation of these repressive marks, a process catalyzed by the age-dependent accumulation and coordinated action of active DNA glycosylases, such as the *DML* family [43, 44]. This fundamental shift in the balance between methylation maintenance and active removal generates a permissive chromatin state, granting the transcriptional machinery access to previously silenced *MADS-box* TF loci [45, 46]. Furthermore, our evidence supports the hypothesis that dense intronic and gene-body CHG methylation functions as a physical barrier to transcription elongation; its localized removal represents a critical prerequisite for the efficient progression of RNA polymerase II through large and complex developmental genes [47–49]. By utilizing intronic CHG methylation as a reversible and cumulative epigenetic memory, woody species can effectively delay the floral transition until sufficient biomass and metabolic energy reserves are secured [50]. This elegant coordination between DNA methylation landscapes and internal developmental cues represents a vital evolutionary adaptation for balancing vegetative longevity with reproductive success within forest ecosystems.

DNA methylation functions as a fundamental epigenetic architect in plants, modulating gene expression across diverse genomic landscapes, including promoters, gene bodies, and repetitive sequences. Our WGBS in *P. chekiangensis* demonstrates that while CG methylation remains remarkably stable across distinct developmental stages, likely acting as a constitutive epigenetic safeguard to ensure genome integrity and the persistent silencing of transposable elements [51–53], the non-CG contexts exhibit profound developmental plasticity. Specifically, we observed a progressive, age-dependent attenuation of CHG methylation, particularly within gene bodies, which potentially facilitates chromatin decondensation and the synchronized activation of master developmental regulators [54, 55]. Further subcontext analysis revealed a sophisticated spatial specialization of methylation: while CAG and CTG sites are ubiquitously distributed across functional regions, CCG sites are uniquely enriched in centromeric regions. This spatial bias potentially provides a structural adaptation to stabilize centromeres within transposon-dense genomic environments [39, 56, 57]. A cornerstone of this study is the mechanistic elucidation of *PcMADS19.1* regulation via site-specific epigenetic remodeling. We found that intronic CHG methylation of *PcMADS19.1* decreases significantly with tree maturity, displaying a robust negative correlation with its transcript abundance. Detailed targeted bisulfite sequencing combined with 5-azaC chemical treatments confirmed that high intronic methylation during juvenile stages functions as a potent repressive barrier. These findings highlight a refined regulatory mechanism wherein gradual, targeted demethylation serves as the primary epigenetic switch for the transcriptional activation of floral integrators. Ultimately, this establishes a previously uncharacterized link between localized DNA methylation dynamics and the age-dependent floral transition in Lauraceae.

**Figure 7:**
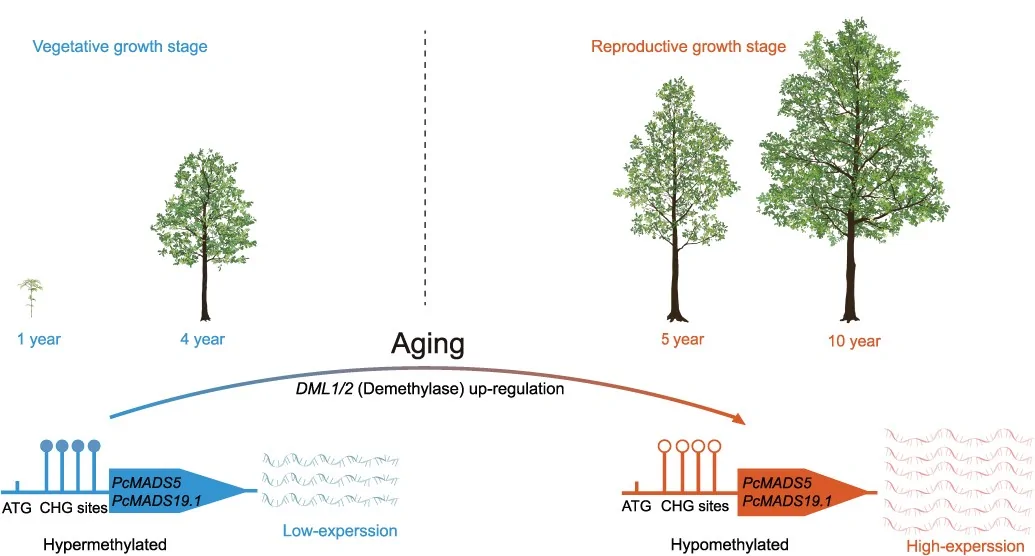
Schematic model of age-dependent intron demethylation-mediated activation of *PcMADS19.1*. High intron methylation maintains low *PcMADS19.1* expression during the juvenile phase, preventing flowering. Progressive demethylation with age activates *PcMADS19.1* transcription, thereby promoting floral transition.

Building upon these comprehensive findings, we propose a mechanistic working model wherein the intronic region of *PcMADS19.1* is maintained in a hypermethylated state during the extended juvenile phase, effectively repressing its transcription. As the tree matures, this specific locus undergoes progressive, age-dependent demethylation concomitant with robust transcriptional activation, thereby driving the vegetative-to-reproductive phase transition in *P. chekiangensis*. Ultimately, this paradigm positions the dynamic intronic methylation of *PcMADS19.1* as a fundamental epigenetic determinant dictating the precise timing of the floral transition (Fig. 7).

## Methods

### Plant materials

All plant materials of *P. chekiangensis* utilized in this study were harvested in May 2024 from the Qinyuan Forest Farm (Qingyuan County, Zhejiang, China). Immediately upon collection, all tissue samples were flash-frozen in liquid nitrogen and permanently stored at −80°C until subsequent nucleic acid extraction. To construct the reference genome, a vigorous, ~10-year-old *P. chekiangensis* individual was selected for whole-genome sequencing (WGS). Notably, this identical tree served as the biological source for the 10-year-old leaf samples used in the WGBS. For the WGBS epigenetic profiling, leaf tissues were systematically collected across four distinct developmental stages (1-, 4-, 5-, and 10-year-old trees), comprising three independent biological replicates per stage. For the full-length transcriptome sequencing, five distinct tissue types (roots, stems, leaves, flowers, and xylem) were similarly harvested in biological triplicates. For the exogenous epigenetic modification assays, vegetative branches excised from 4-year-old trees were hydroponically maintained in a nutrient solution under strictly controlled environmental conditions. These branches were treated with 50 μM 5-azaC via sequential foliar spraying at 3-day intervals over a total period of 15 days.

### Library preparation and sequencing

Single-molecule real-time (SMRT) sequencing libraries were constructed strictly following the standard Pacific Biosciences protocol. High-molecular weight genomic DNA was extracted utilizing the QIAamp Fast DNA Tissue Kit (Qiagen, Germany), mechanically sheared to ~20 kb using g-TUBEs (Covaris, USA), and subsequently subjected to damage repair, end repair, and the ligation of stem-loop adapters. Precise size selection was performed using the BluePippin system (Sage Science, USA), and the resulting fragment distributions were verified via an Agilent 2100 Bioanalyzer (Agilent Technologies, USA). The finalized SMRTbell libraries were generated through the ligation of hairpin adapters and sequenced on the PacBio Revio platform.

For Oxford Nanopore ultra-long-read sequencing, DNA concentration was accurately quantified via a Qubit fluorometer. The extracted DNA was enzymatically repaired utilizing the NEBNext FFPE DNA Repair Mix (NEB, E7360) and the NEBNext Ultra II End Repair/dA-Tailing Module (NEB, E7546). Following adapter ligation, quality assessment, and tethering, the prepared ultra-long libraries were sequenced on a PromethION P48 platform.

Chromosome conformation capture (Hi-C) libraries were prepared by initially subjecting the plant cells to formaldehyde cross-linking, followed by chromatin digestion with the *Dpn*II restriction enzyme. The resulting sticky ends were repaired and labeled with biotin prior to the proximity ligation of physically adjacent DNA fragments. Following the reversal of the cross-links, the genomic DNA was extracted, mechanically sheared, and specifically enriched for biotin-labeled fragments. The final Hi-C libraries (~300 bp) were subjected to paired-end sequencing utilizing the MGI T7 platform.

For whole-genome DNA methylation profiling, genomic DNA was fragmented and subjected to bisulfite conversion, followed by sequential library preparation utilizing the Accel-NGS Methyl-Seq Kit (Swift Biosciences, USA). The resulting libraries were amplified via indexed PCR, and residual oligonucleotides alongside short DNA fragments were rigorously depleted using solid-phase reversible immobilization (SPRI) magnetic bead cleanups. The final methylome libraries were sequenced in a 2 × 150 bp paired-end format on an Illumina HiSeq 4000 platform. Crucially, this robust sequencing effort achieved a bisulfite conversion efficiency exceeding 98% and provided >80% spatial coverage for genomic cytosines at a sequencing depth of ≥4 reads per site.

### *K*-mer analysis and genome size estimation

A *k*-mer frequency analysis (*k* = 17) was performed to estimate the haploid genome size and evaluate the degree of heterozygosity utilizing the Illumina WGS dataset. Prior to analysis, rigorous quality control was implemented, encompassing the removal of unpaired reads, low-quality sequences, adapter contamination, and PCR duplicates. Following these filtering steps, *k*-mers were systematically extracted from the high-quality processed reads. The resulting *k*-mer frequency distribution for *P. chekiangensis* exhibited a distinct bimodal profile, with characteristic peaks observed at 17× and 34×, thereby deviating from the canonical Poisson distribution expected for homozygous genomes. Based on the standard formula (total *k*-mer count / expected *k*-mer depth), the haploid genome size was estimated to be 1 001 973 621 bp. Furthermore, comparative curve fitting against *A. thaliana* heterozygosity gradients indicated an estimated genomic heterozygosity rate of 3.5%, which is highly consistent with the prominent heterozygous peak identified in the *k*-mer profile.

### Genome assembly and validation

We integrated PacBio HiFi reads, Oxford Nanopore Technologies (ONT) ultra-long reads, and Hi-C data to perform a comprehensive hybrid genome assembly. The computational workflow encompassed error correction, the construction of multiplex de Bruijn graphs, and the strategic alignment of ONT reads to resolve complex repetitive regions and complex topological entanglements. Initial draft assemblies were generated using *hifiasm* (v0.19.5; parameters: -s 0.5 --path-min 0.2 --path-max 0.9 -y 0.2 -x 0.98 --ul-cut 100 000) and *Verkko* (v2.1; parameters: --min-ont-length 100 000 --no-correction --haplo-divergence 0.12) for comparative evaluation [58]. The *Verkko* assembly exhibited superior contiguity metrics (N50 and N90) and was consequently selected for all downstream refinement [59].

Chromosome-level scaffolding was achieved utilizing Hi-C proximity ligation technology. Hi-C reads were aligned to the draft contigs using *Chromap* (v0.2.6; parameters: --preset hic --remove-pcr-duplicates) [60]. Subsequently, the contigs were scaffolded into 24 pseudochromosomes using *YaHS* (v1.2; parameters: -e GATC --no-contig-ec) [61]. Chromatin interaction patterns were visualized and manually curated through heatmap generation with *Juicebox* (v1.11.08) [62].

To eliminate remaining genomic gaps, we implemented the *GapFiller* module within the *quarTeT* toolkit (v1.2.5), which integrated the *hifiasm*-derived assembly results to close interstitial gaps [63]. Specifically, this targeted refinement process addressed gap regions on chromosomes 1 and 10 in Pch_hap1, as well as chromosome 1 in Pch_hap2, utilizing the parameters: -f 10 000 -l 1000 -m 10 000 000.

To achieve complete T2T status, we implemented a dual-approach strategy to resolve terminal sequences. First, the *nucmer* module from *MUMmer4* (v4.0.0beta2; parameters: --mum -c 100 -b 500 -l 50) was utilized to align chromosome termini lacking canonical telomeric repeats against alternative assembly versions to identify supplementary sequences [64]. Concurrently, ONT ultra-long reads were anchored to the genome using *Minimap2* (v2.17; parameters: -x map-ont -r 10 000 -N 50 -a) to extract terminal reads spanning the missing telomeric regions [65]. Through this integrated methodology, we successfully generated gap-free T2T assemblies by completing the 3′ end of chromosome 8 and the 5′ end of chromosome 10 in Pch_hap1, alongside the 5′ ends of chromosomes 1 and 8, and the 3′ ends of chromosomes 9 and 10 in Pch_hap2.

### Validation of genome assembly

The final genome assembly was comprehensively validated utilizing multiple high-stringency quality assessment methodologies. Assembly completeness was rigorously evaluated using *Compleasm*, which executes an optimized *BUSCO* analysis against the eudicots_odb10 lineage-specific database. Base-level consensus accuracy and assembly redundancy were estimated via *Merqury* by comparing *k*-mer spectra between the finalized assembly and the unassembled high-quality Illumina reads; a QV >30 was achieved, corresponding to a base-calling accuracy of at least 99.9%. To specifically assess the assembly integrity of complex repetitive regions, the *LAI* was calculated using *LTR_retriever*, which quantifies the proportion of intact LTR-RTs relative to the total identified LTR sequence length. The resulting *LAI* values significantly exceeded the gold standard threshold (LAI >20), thereby confirming the high-fidelity reconstruction of repetitive genomic landscapes in *P. chekiangensis*.

### Gene prediction and functional annotation

Protein-coding genes were predicted using a comprehensive pipeline integrating transcriptome-based, homology-based, and *de novo* computational methods. For transcriptome-supported prediction, Illumina RNA-seq and PacBio Iso-seq datasets were processed independently. RNA-seq reads were aligned to the reference genome to define splice junctions and initial gene structures, while Iso-seq analysis yielded 242 852 high-quality full-length transcripts. Both transcriptomic datasets were subsequently utilized by *PASA* to generate and refine high-fidelity gene models.

For homology-based annotation, protein sequences from representative plant species including *P. bournei*, *Populus trichocarpa*, *Vitis vinifera*, and *A. thaliana* were aligned to the *P. chekiangensis* genome to facilitate the structural annotation of orthologous genes. Additionally, *de novo* gene prediction was performed using *AUGUSTUS* specifically configured with *Arabidopsis*-trained parameters.

Predictions from all three independent strategies were integrated into a consensus gene set using *EVM*. The relative evidence weights were prioritized in the following order: Iso-seq/*PASA*, RNA-seq/*PASA*, Homology, and *de novo*. The resulting integrated gene set was further categorized into HC and LC subsets based on the strength of supporting transcriptional evidence.

Functional annotation of the consensus gene set was conducted through a multifaceted approach. Protein domains and metabolic pathways were characterized using *InterProScan*, while sequence similarities were assessed via *DIAMOND* searches against the UniProt and NCBI Non-Redundant (NR) databases. Functional classifications including GO terms and KEGG pathways were systematically assigned using *eggNOG-mapper*. Furthermore, the gene models were benchmarked against the *Arabidopsis* proteome to infer potential functional orthology. Collectively, this integrated strategy provided a robust and comprehensive functional landscape of the *P. chekiangensis* gene set.

### Repeat sequences annotation

Repetitive sequences were identified using an integrated strategy combining *de novo* and homology-based computational approaches. Initially, a *de novo* repeat library was constructed to serve as a custom reference for subsequent genome-wide annotation. For the detailed characterization of LTR-RTs, a comprehensive pipeline incorporating multiple LTR prediction algorithms was employed to generate a nonredundant LTR-RT library for definitive genome annotation. Repetitive content statistics were derived from the final genome-wide annotation. Full-length LTR sequences including *Unclassified*, *Gypsy*, and *Copia* elements were systematically analyzed to determine their structural characteristics. These analyses encompassed the calculation of length distributions and genomic proportions of each repetitive class. The insertion times of LTR-RTs were estimated based on the sequence divergence between the 5′ and 3′ LTRs of each intact element, utilizing a standard plant synonymous substitution rate to perform molecular dating of transposition events.

### Telomere and centromere detection

Telomere sequence identification was performed utilizing the *teloexplorer* module of *quarTeT* to scan for canonical repeat units throughout the genome. The most abundant candidate sequences were quantified and their distributions were visualized across the genome using 10 kb sliding windows. For centromere localization, the *Centro Locate* tool within the *TBtools* platform was employed with the following specific parameters: a minimum repeat length of 100 bp, a maximum repeat length of 200 bp, a window size of 5000 bp, and a step size of 1000 bp. This analysis facilitated the identification of genome-wide tandem repeats and their constituent monomers. Candidate centromeric sequences were subsequently derived based on the density and distribution patterns of these tandem repeats across each chromosome.

### Circle of genomic landscape

Gene density, repetitive sequence density, and GC content were calculated utilizing custom *Python* scripts with a 1 Mb sliding window across the genome. The resulting data files, which encompassed chromosome identifiers, genomic coordinates, and respective metric values, were subsequently integrated into the *shinyCircos-V2.0* platform for visualization. Key graphical parameters, including track indices, color schemes, and *y*-axis baseline coordinates, were systematically configured to generate the final circular genomic landscape plot.

### Phylogenetic analysis

Centromeric monomer sequences were aligned using *MUSCLE*, followed by maximum-likelihood phylogenetic reconstruction utilizing *RAxML*. The resulting phylogenetic trees were visualized and annotated through the *iTOL* platform.

For the analysis of LTR-RTs, full-length LTR sequences derived from the *LTR_retriever* output and incomplete sequences identified by *RepeatMasker* were randomly sampled. Sequences exhibiting sequence divergence >15% were excluded from the dataset, and fragments of ~2000 bp were selected for downstream analysis. These sequences were subjected to the same alignment and phylogenetic reconstruction workflows as described above for the centromeric monomers.

In the multispecies comparative framework, single-copy orthologs identified by *OrthoFinder* across 10 plant species were aligned and filtered to ensure high-quality data. Concatenated alignments, encompassing all missing sites, were subsequently analyzed using *RAxML* for maximum-likelihood phylogenetic reconstruction to resolve the evolutionary relationships among the sampled taxa.

### GO enrichment analysis

To identify genes associated with unclassified LTR elements, genomic overlaps between genes and LTR sequences were determined utilizing ***BEDTools***. The corresponding GO annotations were subsequently extracted for the identified gene sets. GO enrichment analysis was performed utilizing the ***topGO*** R package, with the complete set of annotated *P. chekiangensis* genes serving as the background. Parallel GO enrichment analyses were executed for two additional gene categories, specifically genes intersecting with structural variation (SV) breakpoints and those situated within nonaligned genomic regions. All enrichment computations were conducted utilizing default algorithmic parameters to ensure consistency across the analyses.

### Genome comparisons and SV identification

Whole-genome alignment between the Pch_hap1 and Pch_hap2 haplotypes was conducted utilizing the *nucmer* module within the *MUMmer* software package. Subsequent to the initial alignment, pairwise syntenic blocks were identified and converted into the standardized format required for downstream analysis. Structural variations were then systematically detected and visualized utilizing the *SyRI* pipeline to characterize genomic differences and rearrangements between the two haplotypes.

### Whole-genome bisulfite sequencing and data analysis

For WGBS library preparation, high-quality genomic DNA was fragmented via ultrasonication. Size selection was performed utilizing 0.7–1.2 LC magnetic beads. The resulting DNA fragments were subjected to bisulfite conversion utilizing the *EZ DNA Methylation-Gold Kit*. Bisulfite converted DNA was ligated to 3′ adapters, followed by second-strand synthesis and purification with 1.2 LC beads. Subsequently, 5′ adapters were ligated, and a final purification step was conducted using 1.0 LC beads. Libraries were amplified using index primers for 10–19 cycles, with the exact cycle number determined by the initial DNA input. A final cleanup was performed utilizing 0.8 LC beads. Unmethylated lambda DNA at a concentration of 0.5 per mille was supplemented as an internal control to evaluate bisulfite conversion efficiency. Library concentrations were accurately quantified utilizing a *Qubit* fluorometer.

Raw sequencing data were processed utilizing *Trimmomatic* (v0.40) for adapter removal and quality filtering [66]. The reference genome was indexed and reads were aligned utilizing *Bismark* (v0.22.3) in conjunction with *Bowtie2* [67]. Alignment parameters were configured with a seed length of 20 bp and zero permissible mismatches. Unmapped and multimapped reads were retained in the initial processing, while duplicate reads were subsequently removed utilizing *deduplicate_bismark*. Only cytosine residues with a sequencing depth exceeding five reads per biological replicate were retained for downstream analysis. Single base methylation levels were extracted utilizing the *bismark_methylation_extractor*. The genome was partitioned into nonoverlapping 1-Mb intervals, and the average methylation level was calculated for each discrete window. *ViewBS* (v0.1.11) was utilized to extract specific methylation profiles from promoters, gene bodies, and other genomic features [68]. Statistical data visualization was performed utilizing the *ggplot2* package (v3.5.0) within the *R* programming environment (v4.3.1) [69].

### RNA-seq and data analysis

Total RNA was extracted utilizing *TRIzol* reagent (*Thermo Fisher*, 15 596 018) strictly following the manufacturer instructions. RNA concentration and purity were quantified utilizing a *NanoDrop ND 1000* spectrophotometer (*NanoDrop*, USA), while RNA integrity was evaluated utilizing a *Bioanalyzer 2100* system (*Agilent*, USA). Only RNA samples characterized by concentrations exceeding 50 ng per μl, RIN values >7.0, and a total RNA mass >1 μg were utilized for downstream library construction. Messenger RNA (mRNA) was isolated utilizing oligo dT magnetic beads (*Dynabeads Oligo dT*, *Thermo Fisher*, USA) and fragmented via the *NEBNext Magnesium RNA Fragmentation Module* (*NEB*, USA). First- and second-strand cDNA synthesis were performed utilizing *SuperScript II Reverse Transcriptase* (*Invitrogen*, USA). The resulting double-stranded cDNA libraries were processed and sequenced on the *Illumina NovaSeq 6000* platform in a 150-bp paired-end format. Highly variable genes across all samples were identified for downstream expression profiling. The mean and standard deviation of expression levels were calculated for each gene, and those exceeding a predefined threshold of variability were retained for further analysis. These selected genes were subsequently partitioned into distinct coexpression patterns utilizing the *k* means unsupervised clustering algorithm.

### Plasmid construction and transformation of *A. thaliana*

For the construction of the overexpression vector, the full-length coding sequence of the target gene was cloned and recombined into the *PK2GW7* expression vector, which is driven by the *35S* promoter and contains an *eGFP* selection marker. Sequence-verified recombinant plasmids were introduced into *Agrobacterium tumefaciens* strain *GV3101* and subsequently transformed into *A. thaliana* utilizing the floral dip method. Transgenic plants were identified based on their resistance to Murashige and Skoog (MS) medium supplemented with kanamycin and the presence of the *eGFP* marker visualized under ultraviolet (UV) illumination. Positive transgenic lines were propagated to the *T3* generation to ensure genetic stability for all subsequent experimental procedures. Selected transgenic plants were cultivated under long-day conditions consisting of a 16 h light and 8 h dark photoperiod at a constant temperature of 23°C.

### RNA extraction and qRT-PCR

Plant tissues stored at –80°C were rapidly transferred to liquid nitrogen and pulverized into a fine powder. Total RNA was extracted utilizing the *FastPure Universal Plant Total RNA Isolation Kit* (*Vazyme*, Nanjing, China). The extracted RNA was subsequently reverse-transcribed into cDNA utilizing the *HiScript III QRT SuperMix for qRT PCR with gDNA wiper* kit (*Vazyme*, Nanjing, China). Specific primers were designed utilizing the *Primer3* web interface and synthesized as detailed in Table S19. qPCR analysis was performed utilizing *ChamQ SYBR Color qRT PCR Master Mix* (*Vazyme*, Nanjing, China) on a *Bio Rad CFX 96* real-time PCR detection system (*Bio Rad Laboratories*, USA).

## Supplementary Material

Web_Material_uhag171

## Data Availability

Supplementary Information is available online. The sequence data generated in this study have been deposited in the China National GeneBank DataBase (CNGBdb) under the accession number CNP0008181. The data supporting the main findings of this study are deposited in the FigShare database (https://doi.org/10.6084/m9.figshare.30338869).
