# Supplementary material for: Telomere-to-telomere genome of Phoebe chekiangensis reveals that age-dependent CHG hypomethylation promotes floral transition via MADS-box gene activation
Source: Hortic Res. 2026 Apr 24;13(9):uhag171. doi: 10.1093/hr/uhag171 (PMC13537828; doi:10.1093/hr/uhag171)
Supplement: Supplementary file 1 [file web_material_uhag171.zip › SupplementInfo.docx]

**Supplement information**

**Telomere-to-telomere genome of *Phoebe chekiangensis* reveals age-dependent CHG hypomethylation promoting floral transition via MADS-box gene activation**

Qinglin Sun ^1, 2, #^, Baoan Wang^1, 2, #^, Yuting Zhang^1, 2, #^, Xiao Han ^1, 2^, Qi Yang ^1, 2^, Zaikang Tong ^1, 2,^ *, Junhong Zhang ^1, 2,^ *

^1^ State Key Laboratory for Development and Utilization of Forest Food Resources, Hangzhou, 311300, China

^2^ Zhejiang Key Laboratory of Forest Genetics and Breeding, Zhejiang A&F University, Hangzhou, 311300, China

***Corresponding authors**

Zaikang Tong, Junhong Zhang

Zhejiang A&F University, 666 Wusu Street, Lin’an, Hangzhou, 311300, Zhejiang, China

Tel.: +86 571 63743855; Fax: +86 571 63743855;

E-mail addresses: [zktong@zafu.edu.cn](mailto:zktong@zafu.edu.cn), [zhangjunhong@zafu.edu.cn](mailto:zhangjunhong@zafu.edu.cn)

^#^ Qinglin Sun, Baoan Wang and Yuting Zhang contributed equally to this study.

**Figure S1** K-mer depth distribution in *P. chekiangensis.* The 17-mer frequency distribution exhibited a characteristic bimodal pattern, with primary and secondary peaks at depth of 17× and 34×, respectively. The genome size was estimated to be 1,001,973,621 bp based on this distribution. The red curve represents the computationally fitted K-mer frequency distribution at 34× depth with 3.5% heterozygosity, simulated using the *Arabidopsis thaliana* reference genome.

**Fig. S2** Hi-C interaction heatmap of *P. chekiangensis*. Chromosome-scale scaffolding was performed based on chromatin interaction data, using Chromap and Yahs.

**Figure S3** BUSCO assessment of Pch_hap1 and Pch_hap2. Genome completeness was assessed against the eudicot_odb10 database, which contains 2,326 single-copy orthologs. Complete BUSCOs are categorized as single-copy or duplicated.

**Figure S4** Telomere organization in *P. chekinngensis* chromosomes. All chromosome termini were capped by telomeres. Distinct telomeric repeat motifs were identified between haplotypes: "AAACCCT" in Pch_hap1 and "AAATGAG" in Pch_hap2.

**Fig. S5** Centromeric regions in *P. chekiangensis*. Distribution of tandem repeats and predicted centromeric regions on chromosomes of (A) Pch_hap1 and (B) Pch_hap2.

**Fig. S6** Phylogenetic analysis of centromeric monomer sequences. A maximum-likelihood phylogeny was constructed for centromeric monomer sequences from *P. chekiangensis*, using the *Arabidopsis thaliana* monomer as an outgroup. The sequences form two distinct classes (Class I and Class II).

**Fig. S7** Characterization and insertion age of major LTR retrotransposons. The donut chart shows the proportion of the number of complete LTR retrotransposons. The pie chart shows the proportion of the total sequence length of complete LTR retrotransposons. The bar chart displays the distribution of insertion times for complete LTR retrotransposons, indicating a recent burst of activity.

**Fig. S8** Exon and intron count analysis across nine angiosperm species. The number of exons and introns per gene shows no significant difference across the analyzed gene sets. Species abbreviations are as defined in Figure 2.

**Fig. S9** SSE-based determination of transcriptome expression clusters. Scree plot depicting the sum of squared errors (SSE) for varying numbers of clusters based on transcriptome expression profiles. The optimal number of clusters was selected at the “elbow” point, where further increases in cluster number resulted in minimal reduction of SSE, indicating an appropriate balance between cluster granularity and overall variance explained.

**Fig. S10** GO enrichment analysis of Cluster 5 genes. Gene Ontology (GO) enrichment analysis of Cluster 5 genes. The analysis revealed significant enrichment in biological processes related to photosynthesis and chloroplast organization, highlighting the functional coherence of genes co-expressed in this cluster.

**Fig. S11** KEGG pathway enrichment of Cluster 5 genes. KEGG pathway enrichment analysis of Cluster 5 genes. Cluster 5 genes were significantly associated with pathways involved in energy metabolism, including carotenoid biosynthesis and glutathione metabolism, as well as ribosome function and protein folding, indicating their coordinated role in cellular metabolic and translational processes.

**Fig. S12** Flowering phenotypes of Arabidopsis overexpressing PcMADS5 and PcMADS19.1. Representative phenotypes of wild-type (WT) plants and transgenic lines are shown at the bolting stage. Quantification of flowering time was assessed by counting the number of rosettes leave at bolting (n ≥ 20 plants per genotype). Data are presented as mean ± standard deviation (SD). Statistical significance was determined using one-way ANOVA followed by Tukey’s HSD post hoc test. Different letters (a, b, c) above the bars indicate statistically significant differences between groups (*P* < 0.05).

**Fig. S13** Phylogenetic relationships and conserved domain architectures of epigenetic regulatory proteins in P. chekiangensis. The phylogenetic tree on the left illustrates the evolutionary relationships among orthologous proteins identified from Arabidopsis thaliana (prefix At) and P. chekiangensis (prefix Pc). The analysis encompasses core components across three major pathways: the DNA methylation maintenance pathway (including MET, CMT, SUVH, VIM, IBM, and DDM families), the RNA directed DNA methylation (RdDM) pathway (including AGO, DCL, RDR, DNMT, and NRPD families), and the active DNA demethylation pathway (the DML family). Conserved domain organizations are displayed on the right, with specific structural motifs identified via color coded boxes as defined in the domain legend. The scale bar at the bottom denotes the protein sequence length in amino acids (aa).

**Fig. S14** Expression trajectories of epigenetic regulatory genes across developmental stages. Unsupervised *k* means clustering of 94 core epigenetic components in *P. chekiangensis* identifies nine distinct expression trajectories (*Cluster 1* to *Cluster 9*). The number of genes assigned to each cluster is specified in parentheses within each panel. Transcriptional profiles were evaluated across four developmental stages, including 1, 4, 5, and 10 year old trees. Relative expression levels (Z scores calculated from *FPKM* values) are color coded as indicated by the scale bar, where red represents upregulation and blue represents downregulation. Individual rows within each heatmap correspond to specific gene members, while columns represent the chronological sampling points.

**Fig. S15** Quality assessment of WGBS data. Assessment of whole-genome bisulfite sequencing (WGBS) data quality, including bisulfite conversion efficiency and sequencing coverage across the *P. chekiangensis* genome. High conversion rates and uniform coverage indicate reliable methylation calling and overall data quality.

**Fig. S16** Distribution and methylation patterns of different CG subcontexts in the *P. chekiangensis* genome. Genome-wide analysis of CG subcontexts (CGA, CGC, CGG, and CGT) showing their relative abundance and DNA methylation levels. This analysis reveals context-specific methylation patterns and provides insights into the regulation of CG methylation in *P. chekiangensis*.

**Fig. S17** Chromosome-level distribution and methylation profiles of CHH subcontexts in *P. chekiangensis*. Chromosomal mapping of CHH subcontexts (CAA, CAG, CAT, CTA, CTC, CTG, CTT, etc.) and their methylation levels across the genome. The profiles highlight variation in CHH methylation across chromosomes and suggest potential regions of epigenetic regulation.

**Fig. S18** Distribution and methylation patterns of different CHH subcontexts in the *P. chekiangensis* genome. Genome-wide distribution and methylation analysis of CHH subcontexts, illustrating context-dependent methylation differences. This figure complements the chromosome-level view by providing a global overview of CHH methylation patterns.

**Fig. S19** Chromosome-level distribution and methylation profiles of CHH subcontexts in *P. chekiangensis*. Detailed chromosome-level profiles of CHH methylation across all identified subcontexts in *P. chekiangensis*, showing the spatial distribution of methylation and potential hotspots of epigenetic modification.
